# The operational window of carbon nanotube electrical wires treated with strong acids and oxidants

**DOI:** 10.1038/s41598-018-32663-0

**Published:** 2018-09-25

**Authors:** S. Lepak-Kuc, S. Boncel, M. Szybowicz, A. B. Nowicka, I. Jozwik, K. Orlinski, T. Gizewski, K. Koziol, M. Jakubowska, A. Lekawa-Raus

**Affiliations:** 1 Faculty of Mechatronics, Warsaw University of Technology, Warsaw, Poland; 2 Department of Organic Chemistry, Bioorganic Chemistry and Biotechnology, Faculty of Chemistry, Silesian University of Technology, Gliwice, Poland; 3 Faculty of Technical Physics, Poznan University of Technology, Poznan, Poland; 4 Institute of Electronic Materials Technology, Warsaw, Poland; 5 Faculty of Electrical Engineering and Computer Science, Lublin University of Technology, Lublin, Poland; 6 Enhanced Composites & Structures Centre, Cranfield University, Cranfield, UK

## Abstract

Conventional metal wires suffer from a significant degradation or complete failure in their electrical performance, when subjected to harsh oxidizing environments, however wires constructed from Carbon Nanotubes (CNTs) have been found to actually improve in their electrical performance when subjected to these environments. These opposing reactions may provide new and interesting applications for CNT wires. Yet, before attempting to move to any real-world harsh environment applications, for the CNT wires, it is essential that this area of their operation be thoroughly examined. To investigate this, CNT wires were treated with multiple combinations of the strongest acids and halogens. The wires were then subjected to conductivity measurements, current carrying capacity tests, as well as Raman, microscopy and thermogravimetric analysis to enable the identification of both the limits of oxidative conductivity boosting and the onset of physical damage to the wires. These experiments have led to two main conclusions. Firstly, that CNT wires may operate effectively in harsh oxidizing environments where metal wires would easily fail and secondly, that the highest conductivity increase of the CNT wires can be achieved through a process of annealing, acetone and HCl purification followed by either H_2_O_2_ and HClO_4_ or Br_2_ treatment.

## Introduction

Carbon nanotube (CNT) fibre conductors are a very interesting new class of materials^1,2^. Their yarn-like, tubular, highly anisotropic and nanostructured, carbon-based morphology makes them intrinsically different to any existing metallic conductors, this potentially paving the way for significant advancements in the performance of existing wiring systems, as well as completely new applications, such as super lightweight electrical machines^3^, smart textiles^4^ or high performance coaxial cables^5^. However one of the quite unusual aspects of CNT wires is their response to oxidants^6–9^.

It is well understood that both copper and aluminum conductors are very sensitive to oxidation. Low levels of oxidation cause the formation of copper/aluminum oxides that are resistive and this oxidization decreases the cross-sectional area of the wires that is available for current transport. Fortunately, passivation of the wires tends to hinder their further oxidation, unless strong oxidants are present in large amounts. For example, dipping both copper and aluminum wires in strong acids causes them to dissolve with a rate dependent on the type, concentration, strength and redox potential of the acid. In order to mitigate these effects more oxidation resistant metals such as gold are used, for example, as electronic bonding wires. But even this metal reacts with aqua regia (HNO_3_ + 3HCl) or chlorine.

On the contrary, CNT fibres have been reported to be structurally resistive to most strong acids and halogens which makes them perfect candidates for applications where metals would fail due to oxidation^10^. Moreover, treating CNT structures with strong acids or halogens, which are known as oxidizing agents, has been seen to cause an increase of their room temperature electrical conductivity^11–17^.

The CNT fibres produced today are composed of both semiconducting and metallic nanotubes. Depending on the fibre, these nanotubes can include those that are short, defective, contaminated, or not densified and aligned perfectly. These issues currently hinder the achievement of the ultimate conductivity predicted for CNT fibres^18^. However, it is also possible to optimize the fibre properties by doping. Taking into account the fact that some degree of p-doping can take place in air^19,20^, this type of doping has been proposed as a promising way of boosting the conductivity^13–17,21–23^.

Although this approach to conductivity improvement is not devoid of issues e.g. as we have shown previously, doped CNT wires can release part of the oxidizing agent upon current flow induced heating^22^. Additionally, since the thermal failure of CNT fibres originates from their rapid oxidation in air, one might expect that the presence of strong oxidants within the wires will influence their failure temperature.

However, the tremendous potential of being able to produce highly conductive oxidation resistant wires led us to perform a comprehensive investigation of the interaction of CNT wires with a wide range of strong oxidants. We conducted a detailed analysis of various doping effects on the CNT wires and a determined the operational window for oxidized CNT wires.

## Methods

### CNT wires

Samples of the CNT material used in this research were produced via the floating catalyst chemical vapor deposition (CVD) method described previously^24,25^. Methane, thiophene and ferrocene provided the feedstock for the production. The CNT wires were prepared via a process utilizing the mechanical rolling of 1 cm wide CNT films, this process facilitated the formation of tightly packed wires of several hundred micrometers in diameter. The samples were then cut to 3–7 cm lengths, depending on the test performed.

### Sample doping and modification

The following list of chemical compounds were used in the purifying and doping treatments: acetone (C_3_H_6_O), toluene (C_7_H_8_), chloroform (CHCl_3_), *n*-hexane (C_6_H_14_)_,_ methanol (CH_3_OH), hydrogen peroxide (H_2_O_2_ (60%)), acetic acid (CH_3_COOH), formic acid (HCOOH), trichloroacetic acid (CCl_3_COOH), hydrochloric acid (HCl (35–38%)), sulfuric acid (H_2_SO_4_ (98%)), nitric acid (HNO_3_ (65%)), perchloric acid (HClO_4_ (70%)), chlorosulfonic acid (HSO_3_Cl (97%)), 20% oleum, I_2_, Cl_2_ (obtained via the reaction of KMnO_4_ with HCl_aq_), ICl, and Br_2_.

All acid treatments were conducted by soaking the samples for 24 hours. ICl and Br_2_ were added by droplet, and I_2_ and Cl_2_ were applied via vapor deposition. The mentioned compounds were purchased from; Linegal Chemicals (C_3_H_6_O, C_7_H_8_, CHCl_3_, C_6_H_14_, CH_3_OH, H_2_O_2_, CH_3_COOH, HCOOH, CCl_3_COOH, HCl, H_2_SO_4_, HNO_3_, HClO_4_, KMnO_4_) Sigma-Aldrich (ICl, oleum), Acros Organics (HSO_3_Cl), ichemia.pl (I_2_) and REKO PR-W (Br_2_). (All of the chemicals were applied as purchased).

The samples were annealed in air using a laboratory muffle furnace Czylok FCF 7 SHM and the samples were sonicated in acetone using Utrasonix Proclean 2.0 M sonication bath.

### Sample characterization

Measurements of the CNT wire diameter were made on a digital optical microscope Keyence VHX-900 F. Weighing was performed on Radwag Ultra-Microbalance UYA 2.4Y. The electrical resistance measurements were conducted using the “two-point method” using a True RMS multimeter UNI-T UT804. Connections between electrodes and CNT wires were facilitated by the use of a silver conductive paint.

The current carrying capacity measurements were performed with a setup specifically designed for this activity comprising a DC power supply TTi QL564P, and a DC Keithley 2000 multimeter, all controlled via proprietary software written in LabVIEW.

Thermal analysis (simultaneous thermogravimetric analysis - TGA) was performed on an STA 449 F1 Jupiter (NETZSCH) apparatus. A standard platinum crucible was loaded with 1.4–2 mg pieces of a carbon-nanotube fabric with these flattened to improve the heat transfer between the sample and the crucible. The measurement was conducted with a heating rate of 5 K/min, under a flow of “synthetic air” (Ar - 72 ml/min, O_2_ - 18 ml/min), from room temperature up to 800 °C.

The Raman scattering spectra of samples were investigated in the spectral range of 100 cm^−1^–3600 cm^−1^. The nonpolarized Raman spectra were recorded via their back scattering geometry using an inVia Renishaw micro-Raman system. For excitation a “light blue” line of argon laser light, operating at 488 nm, was used. The laser beam was tightly focused on the sample surface through a Leica 50x LWD microscope objective (LWD – long working distance) with numerical aperture (NA) equal to 0.5, this leading to a laser beam diameter of approximately 2 μm. Motorized stages were used to perform Raman surface mapping measurements with an area of 40 × 40 µm. Renishaw WiRE 3.4 software was used for deconvolution of the obtained spectra, by the curve fitting method using a mix of Lorentz and Gaussian functions.

The CNT specimens were subjected to Scanning Electron Microscope (SEM) investigations with low energy (less than 5 keV) electrons using the Auriga CrossBeam Workstation (Carl Zeiss) equipped with an In-lens SE (true SE1) detector and an Energy selective Backscattered electron (EsB, low-loss BSE) detector, both positioned on the optical axis of the Gemini (TM) column. In front of the entry system on the axis of the BSE detector there is an energy filtering grid integrated into the electron optical detection system that can be adjusted in its retarding potential from 0 to 1500 volts. This grid allows only BSEs with energies greater than the grid energy to be detected. The energy of the primary electrons was adjusted to effectively reveal all the features of the studied samples in terms of the impurities present within the CNT bundles.

## Results and Discussion

### The influence of oxidative treatments on electrical conductivity of CNT fibres

**Figure 1:**
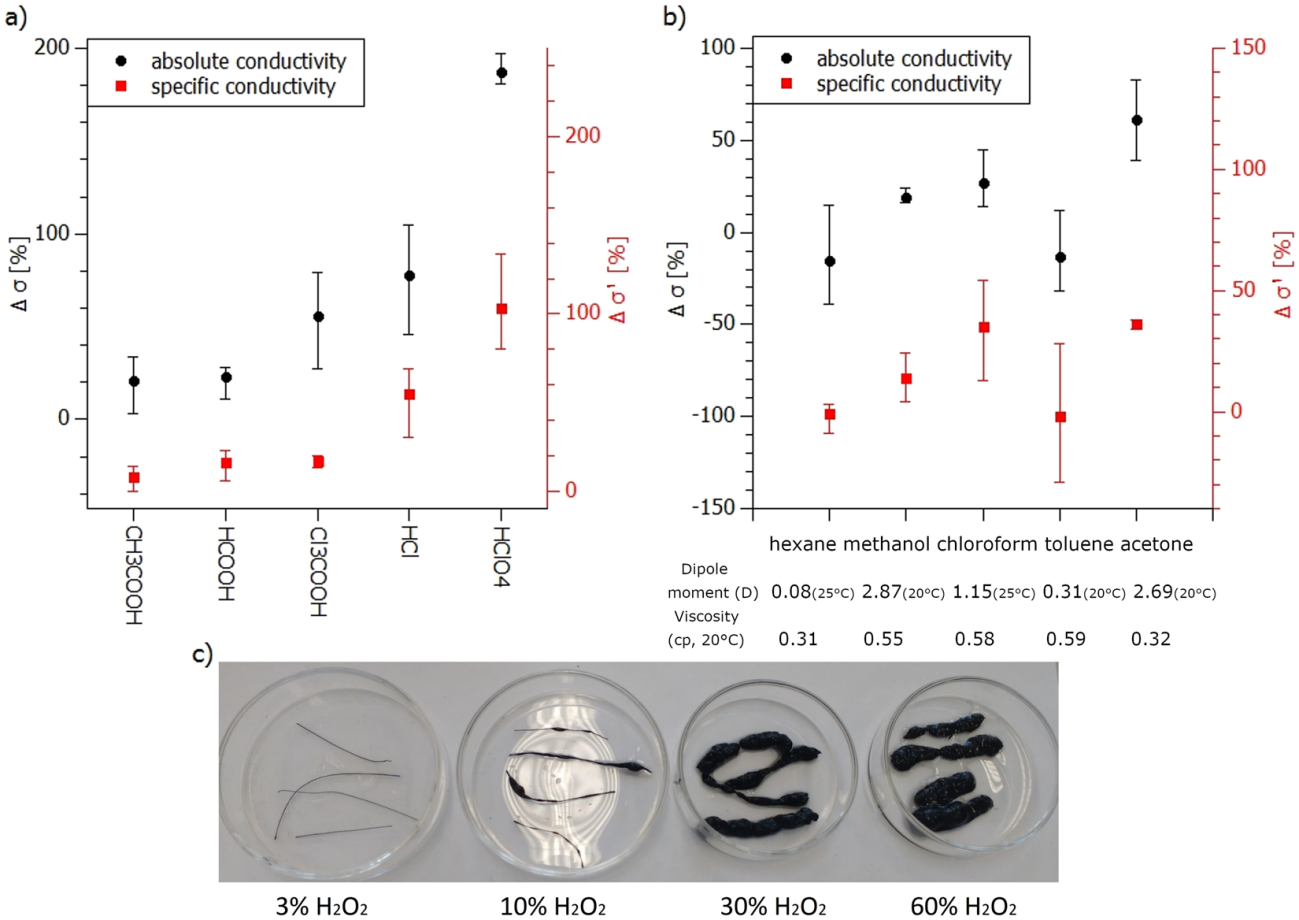
Average conductivity change obtained after treating CNT fiber with (**a**) acids of various acidity (K_a_ in water), (**b**) different organic solvents after pre-annealing. Whiskers represent differences between maximum and minimum values obtained in a series of experiments conducted; (**c**) A photograph illustrating swelling of fibers caused by different H_2_O_2_ concentration.

The influence of acids on CNT fibres may be controlled, not only by their redox potential, but also by the strength of the acid. In order to differentiate between these two factors the initial tests started by treating the fibres with four acids of varying acidity, which at room temperature are non-oxidizing against carbon nanostructures: acetic acid (K_a_ = 1.76 × 10^−5^), formic acid (K_a_ = 1.77 × 10^−4^), trichloroacetic acid (K_a_ = 0.16), hydrochloric acid (K_a_ = 1.3 × 10^6^) and perchloric acid (K_a_ > 10^10^) (Fig. 1a).

After immersion in these acids, the samples – on a macroscopic scale – did not show any visual structural changes. However, as shown in Fig. 1a, the change in their conductivity appears to be related to the strength of the acids with the strongest acid causing the highest conductivity increase. Therefore, while looking for the best conductivity boosting agents and the limits of operation of the doped wires, only the strongest agents including strong acids and halogens were selected for the further tests carried out. However, before moving on to the strong oxidizers tests, one further issue was investigated.

Currently produced CNT fibres/films can contain CVD production derived side-products such as amorphous carbon, hydrocarbons and their derivatives or catalyst particles. Additionally, there is always the small influence of air dopants which may be correlated with the presence of the above impurities^19^. All of these factors can influence the doping effectiveness (in this work defined as the improvement of electrical conductivity) and behavior of CNT wires. Hence, previous research on CNT wire doping often included purification steps, in various configurations^15,26^. The most popular treatments included annealing which was reported to cause the removal of amorphous carbon^21^. Also soaking and sonication in organic solvents, has been reported to remove organic contaminants^27^. While catalyst nanoparticles were supposed to be disposed of, by mineral acids treatment.

In the case of iron-based nanoparticles present in our material (as a catalyst residue formed by ferrocene decomposition in the CVD synthesis process), hydrochloric acid could be indicated as a first choice chemical solvent^22^. There are also reports involving soaking in concentrated hydrogen peroxide as a way to enable the cracking of amorphous carbon and the oxidative dissolution of catalysts particles from the material^15^.

With the aforementioned purification methods considered, a set of tests starting from annealing in air at a range of temperatures was performed.

For this purification step, the greatest increase in both absolute and specific conductivity was recorded for an annealing at 400 °C. Here, the percentage change in absolute conductivity (Δσ) was equal to 18%, while the percentage change in specific conductivity (Δσ*), a measurement which takes into account the weight of the material^1^, was 20%. At a temperature 250 °C, a decrease of conductivity was observed (Δσ = −1%, Δσ* = −1%). And annealing at 500 °C led to a structural disintegration of the wires.

The results indicated that a 400 °C annealing may indeed quantitatively remove amorphous carbon, paving the way for better electron transport in the fibre, while a lower temperature treatment only removes some air dopants.

Following the annealing, further tests involved organic solvent treatments.

The organic solvents chosen for these experiments varied in both polarity and viscosity^28^ (Fig. 1b). After initial annealing, the fibres were subjected to sonication and prolonged soaking in the solvent. It was noted that prolonged sonication periods led to damage of the fibres, but a lack of sonication decreased the effectiveness of the purification process. Therefore, the final procedure was based on a 10 min sonication and 12 hours soaking in the solvents.

As shown in Fig. 1b, all of the solvents had an influence on the electrical properties of the wires with the highest increase in absolute and specific conductivity observed for acetone. These characteristics can be related to the fact that acetone represents a universal solvent of high affinity to aromatic and hydroxy-aromatic compounds (possibly residing after annealing) as well being an efficient densifying CNT fibre agent^29^. Therefore, it was chosen for further experiments.

Following the purification procedure proposed by Zhao *et al*.^15^, the next treatments used were targeted at the removal of catalyst nanoparticles, and these involved soaking in 30% H_2_O_2(aq)_ (48 hours at room temperature) and concentrated HCl_(aq)_ (24 hours at room temperature). It should be noted that the HCl treatment was followed by deionized water rinsing (30 sec long immersion repeated 3 times).

This procedure resulted in an approximately 87% and 95% increase in absolute and specific conductivity, respectively. Interestingly a change in the sequence of these two treatments (HCl followed by H_2_O_2_) resulted in an approximately 117% and 133% increase in absolute and specific conductivity, respectively. This indicates that the conductivity changes observed may be not only due to catalyst removal, but also their doping action.

However, over and above this our observations indicated that the H_2_O_2_ may also have a different and very important role.

It can be seen in Fig. 1c that the soaked samples are visibly swollen, with the magnitude of the swelling related to the concentration of the H_2_O_2_. As shown in Table 1, the use of these different concentrations of H_2_O_2_ had little effect on with absolute and specific conductivity increases but had an observable influence on the effectiveness of the acid doping. The absolute conductivity of the fibre treated with HClO_4_ after 60% H_2_O_2_ soaking was approximately 200% higher than for the fibre treated with HClO_4_ after 3% H_2_O_2_ soaking. While, the specific conductivity (i.e. taking into account the weight of the sample) was increased by approximately 30% only. Both these results lead to the conclusion that the mechanism responsible for the conductivity increase is the increase in the amount of adsorbed acid due to opening/swelling of the structure.

**Table 1:** Percentage conductivity change after purification (annealing, acetone and HCl soaking), purification followed varying concentration H_2_O_2_ treatment, and finally after purification followed by H_2_O_2_ and HClO_4_ treatment. Percentage changes relate to the as-made fibres.

		Δσ [%]	Δσ’ [%]	Δσ [%]	Δσ’ [%]	Δσ [%]	Δσ’ [%]
		purified		Purified + H_2_O_2_		Purified + H_2_O_2_ + HClO_4_	
H_2_O_2_ concentration	3%	167	131	162	135	399	206
	10%	158	130	162	124	402	203
	30%	112	98	117	133	498	205
	60%	126	107	113	136	585	233

Based on this optimization of the absolute and specific conductivity, the purification procedure was recorded and defined as: 1) annealing at 400 °C for one hour, 2) 10 min sonication in acetone with a subsequent soaking for a minimum of 24 hours and 3) soaking in concentrated HCl for 24 hours. Samples doped with acids were also pre-treated by soaking in 60% H_2_O_2_ for 48 hours.

**Figure 2:**
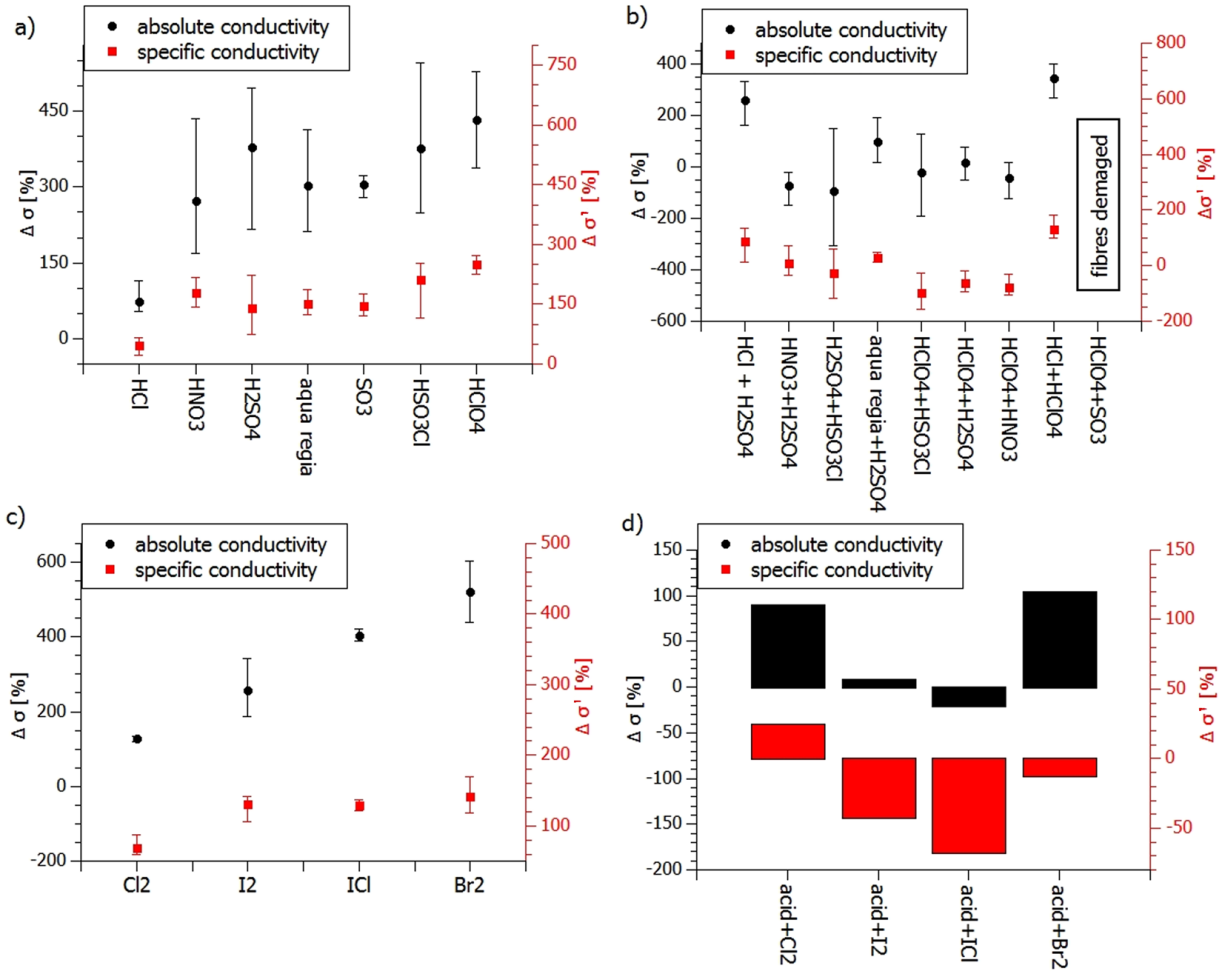
(**a**)Average percentage change in conductivity of wires treated with strong acids, wires were purified and soaked in 60% H_2_O_2_ before acid treatment (**b**) average percentage change in conductivity of wires treated with two strong acids-one after another (difference in conductivity compared to the conductivity after first acid treatment), wires purified and pre-treated with 60% H_2_O_2_ beforehand; (**c**) average percentage conductivity change after halogenation (halogenation performed on purified fibres); (**d**) Average conductivity change after halogenation of CNT fibers pretreated with HClO_4_, after initial purification and H_2_O_2_ treatment. Whiskers represent differences between maximum and minimum values obtained in a series of experiments conducted.

The above pre-treatment procedure was used to condition all of the samples used for further acid doping tests. As mentioned above, only strong acids were used for these experiments and these included H_2_SO_4_, HNO_3_, HClO_4_, HSO_3_Cl, SO_3_, aqua regia (HNO_3_ + 3HCl) and HCl, which is a selection of common, strong oxidizing acids and a strong non-oxidizing mineral acid for comparison. Figure 2a shows the percentage increase in absolute and specific conductivity values after treatment with a given acid. It can be seen that oxidizing acids have a much more significant influence on both the electrical conductivity and specific conductivity of the wires than HCl, which, it is assumed, is related to the doping mechanism induced by these compounds.

However, there is also a physical limit to these doping procedures. Since sulfuric acid and its compositions (oleum) or derivatives (chlorosulfonic acid) are hygroscopic, (additionally chlorosulfonic acid hydrolyzes in humid air to gaseous HCl), hence fibres treated with those reagents disintegrate (individual filaments are repulsed due to strong protonation)^30^.

With a view to finding the further limits of the physical endurance of the samples and checking the possibility of further conductivity boosting, the wires were treated with various combinations of two acids one after the other. Figure 2b shows the percentage change (increase or decrease) in conductivity after the second acid treatment as compared to the results achieved for the first one. Interestingly, the results show that the most effective doping (defined as the increase in conductivity) takes place for HCl + HClO_4_, which are both strong and non-oxidizing acids in water (at room temperature). This result may be explained by the fact that these strong acids dope by a different chemical route, yet they do not damage the fibres physically. Indeed, in acidic aqueous solutions ClO_4_^−^ oxidizes Cl^−^ to chlorine (Cl_2_) since hydronium-doping is enhanced by doping with chlorine (HClO_4_ + 7HCl = 4Cl_2_ + 4H_2_O).

Another set of strong p-dopants includes halogens and interhalogen compounds. To compare the effect of these oxidizers, the purified CNT fibres were treated with vapors or infiltrated with Cl_2_, Br_2_, I_2_ and ICl. The pre-treatment did not involve the H_2_O_2_ step as the pre-tests showed that soaking in hydrogen peroxide had no influence on the effectiveness of halogenation. As shown in Fig. 2c, doping with Br_2_ had the most pronounced effect on the conductivity. This effect could be ascribed to the most time-stable adsorption of bromine on the CNT outer walls and its lower viscosity than for ICl, enabling more efficient penetration of CNT fibres. The moisture-assisted reaction of bromine with water (Br_2_ + H_2_O = 2HBr + [O]) could also yield an additional mechanism of doping based on protonation by strong acid HBr and oxidation by *in situ* formed reactive oxygen. Importantly, none of the halogens caused any visible damage to the wires.

Finally, this halogenation was combined with the most effective HClO_4_ treatment defined above and the results examined. Tests showed that although this combination is not harmful to the structural integrity of the fibres it is also not beneficial to the conductivity (Fig. 2d), causing a decrease in specific conductivity for Br_2_, I_2_ and ICl and only a small increase for Cl_2_.

To summarize briefly, we have defined the most effective doping procedures as purification and 60% H_2_O_2_ soaking, followed by HClO_4_ treatment as well as purification followed by Br_2_ treatment.

The values obtained for the samples amounted to: for HClO_4_ treatment: σ = 8,55∙10^5^ S/m (Δσ = 704%), σ’ = 8,76∙10^2^ S∙m^2^/kg (Δσ’ = 295%); and for Br_2_ treatment: σ = 7,02∙10^5^ S/m (Δσ = 631%), σ’ = 6,51∙10^2^ S∙m^2^/kg (Δσ’ = 158%).

These values are higher than for most CVD spun doped fibres^26,31–33^. The only paper currently reporting higher values, is that mentioned earlier by Zhao *et al*.^15^ in which the authors claim that their doped fibres have higher specific electrical conductivity than copper and aluminium. However, these results were questioned by Behabtu *et al*. (in their supplementary materials)^17^ Our tests, based on the procedures of Zhou *et al*. are also not producing the results predicted in their work. Therefore, there is certainly room for further discussion/research in this area.

### Characterization

#### Scanning electron microscopy (SEM)

**Figure 3:**
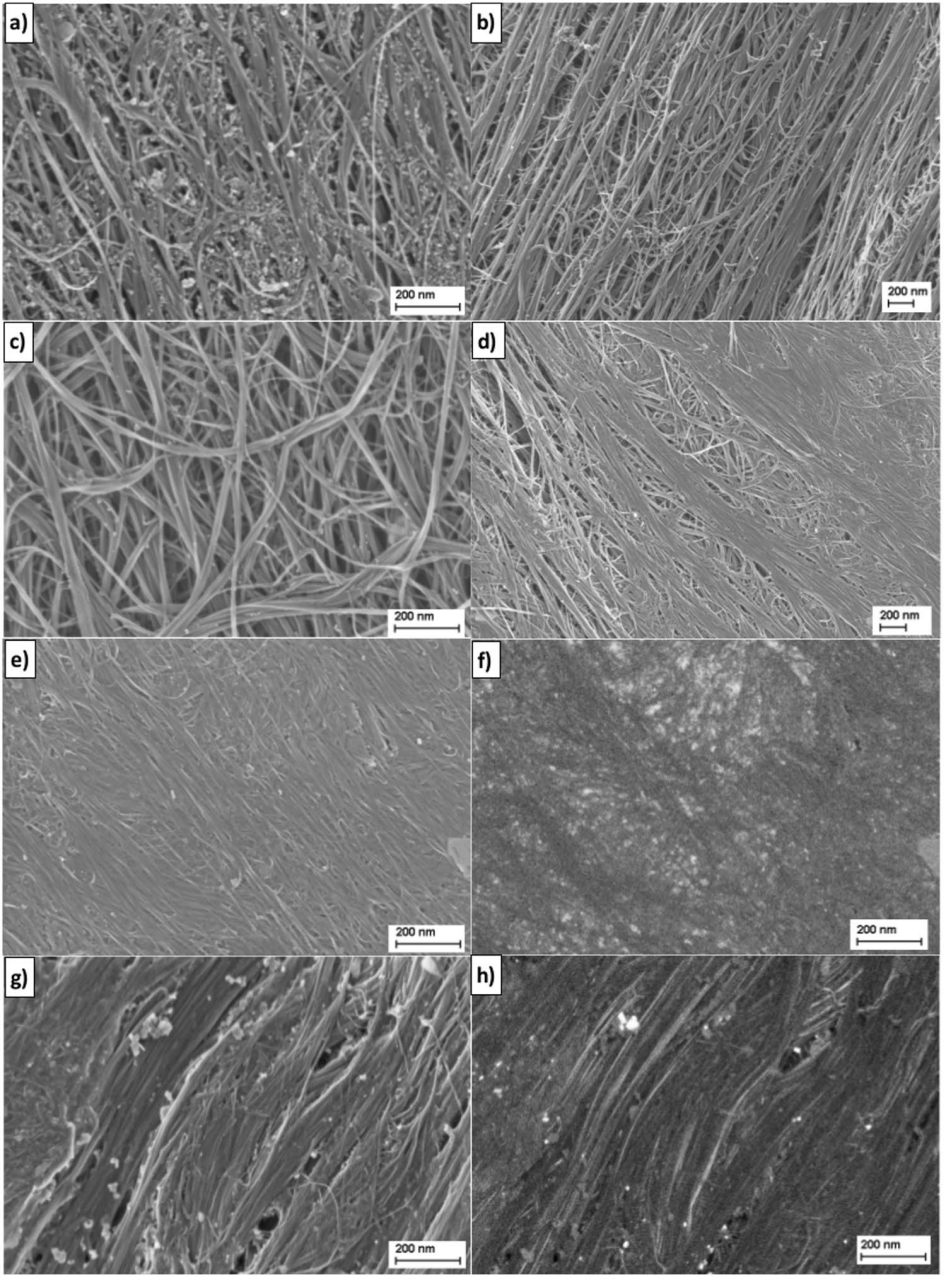
SEM images of: (**a**) CNT fiber contaminated mostly by iron particles; (**b**) Purified fibre; (**c**) Fibre purified and treated with 60% H_2_O_2_ (**d**) Fibre purified, treated with H_2_O_2_ and treated with HClO_4_; Fibre purified and treated with Br_2_ - SE1 image **(e)** and - LL-BSE image (**f**); Fibre purified and treated with I_2_ - SE1 image (**g**) and - LL-BSE image (**h**).

Some of the above results were qualitatively supported by SEM analysis. After the purification process the CNT wires were found to be visibly less contaminated (Fig. 3a,b). Furthermore, CNT fibres swollen by H_2_O_2_ treatment have visibly larger spaces between the CNT bundles at the microscale (Fig. 3c). While, both HClO_4_ and Br_2_ treatments resulted in a densification of the CNT bundles in wires (the effect is more intense after Br_2_ treatment confirming high penetration of the CNT fibre, Fig. 3d,e) and the formation of a uniform smooth coating on the structure, which could ensure the most effective doping with the least weight increase.

For comparison, fibers doped with bromine and iodine differed at the microscale. Separate Br_2_ liquid ‘islands’ are barely visible on the CNT bundles in the SE1 image (Fig. 3e), but they show up clearly as bright spots in the LL-BSE image (Fig. 3f). Larger (up to ca. 50 nm) iodine clusters are clearly visible in the images obtained from both detectors (Fig. 3g,h). Those relatively large and non-uniformly dispersed iodine agglomerates could explain the significant increase in weight which resulted in a decrease in specific conductivity.

#### Thermogravimetric analysis (TGA)

More insight into the changes taking place in the material during the purification and doping procedures was obtained from the thermogravimetric analysis (TGA). This was performed on the samples as they underwent each step of purification and doping, including: 1) samples annealed at 400 °C, 2) samples annealed and treated with acetone, 3) samples annealed, treated with acetone and soaked in HCl, 4) samples annealed, treated with acetone, soaked in HCl and H_2_O_2_, and samples annealed, treated with acetone, soaked in HCl and H_2_O_2_ and doped: with HClO_4_-5a) or H_2_SO_4_-5b).

**Figure 4:**
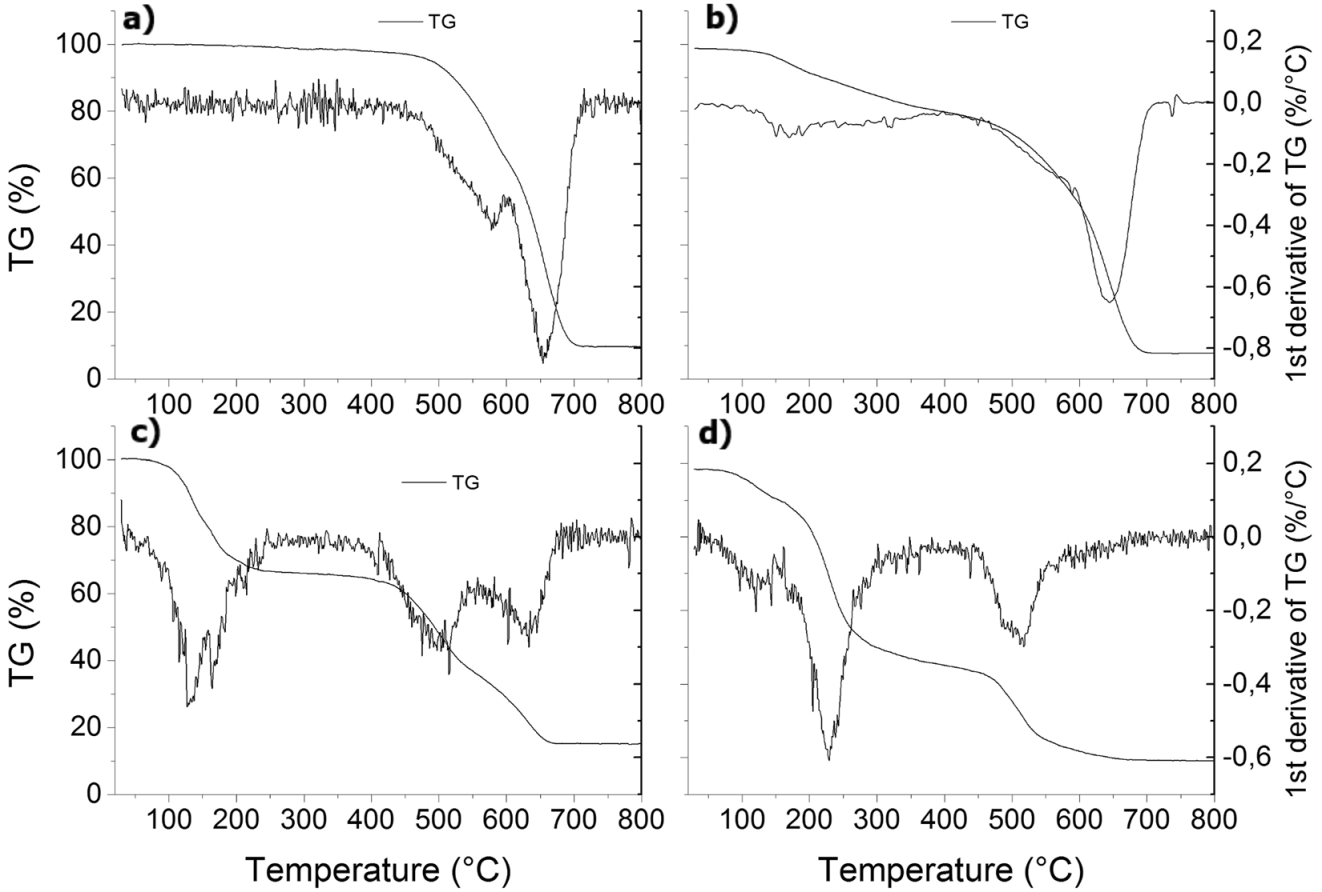
TGA curves for CNT wires: (**a**) after annealing at 400 °C; (**b**) after purification and H_2_O_2_ treatment; (**c**) after purification, pre-treatment with H_2_O_2_ and HClO_4_ treatment; (**d**) after purification, pre-treatment with H_2_O_2_ and H_2_SO_4_ treatment.

Examples of the TGA curves are presented in Fig. 4.

It is clearly visible that the annealed sample, from the outset shows a small steady decrease in mass, which can be related to the air adsorbates (including molecular water) released from the structure. The further decrease in weight may be related to removal of amorphous carbon (temperatures between 250 °C and 400 °C^27^), which is a less graphitized carbon phase than carbon nanotubes, thus having increased reactivity towards oxygen. The weight loss at temperatures between 350 °C and 500 °C is expected to be caused by the removal of hydroxyl functionalities^34^. (Oxidation of well-graphitized structures takes place at temperatures over 500 °C).

The temperature of the elimination of catalyst particles is not straightforward to determine specifically. Both the amounts and microstructures of metal particles could affect the position of the peaks. However, for the undoped structures, the dry matter residue can mostly be associated with the remaining iron oxide catalyst. By analyzing successive TGA graphs, it can be seen that after treating the CNT fibers with HCl the dry matter residue is reduced, which may suggest that the elimination of the catalyst nanoparticles occurred. Comparing the sample after annealing and after HCl treatment, it can be seen that for the annealed sample there is a mass change of 1.4% at temperature ~200 °C, then at 600 °C a mass change of 33.5%, and 55.6% above 600 °C. This gives a total mass change of 90.5%. Thus, the catalyst residue as Fe_2_O_3_ is about 9.5% (hence, the Fe residue is 6.64%). Similar calculations for annealed and acetone and HCl purified sample, give 6.2% and 4.34% for Fe_2_O_3_ and Fe residue respectively.

It can be also observed that the acid treatment caused a reduction in the material stability, which can be related to a drop in the oxidation temperatures^7^. It is also worth noting that the weight drop recorded below ~500 °C was higher for H_2_SO_4_ than for the HClO_4_ treatment (Fig. 4).

This comparison indicates that the samples treated with HClO_4_ contained a higher concentration of well-graphitized material (which means lower content of amorphous carbon or less damaged structures). For the sample doped with perchloric acid the peaks appeared at lower temperatures than for sulfuric acid, which suggest that the CNT wires are well loaded by both acids, and their boiling point (HClO_4_ = 203 °C and H_2_SO_4_ = 338 °C) influences the decomposition of the material.

All the above presented results indicate that the strongly oxidized CNT wires are more sensitive to temperature than their pure/annealed counterparts.

#### Raman spectroscopy

In order to obtain more information on the doped state of CNT wires, a set of Raman spectroscopy measurements were performed, these being a recognised technique for the investigation of the structure and doping state of CNTs.

One of the most important features of CNTs in Raman spectra are: RBM (radial breathing mode) however this is only visible for single wall carbon nanotubes (SWNT) at a sufficiently high concentration. The CNT fibres, tested here, were composed of single-wall, double-wall and multi-wall CNTs in varying concentrations, therefore RBMs were not observable.

Another important feature is D-band (~1350 cm^−1^) which is the result of the double-resonance Raman effect in sp^2^-carbon and corresponds to the presence of amorphous or disordered carbon in the CNT structure^35^. It has been reported that p-doped CNTs respond with an up shift of the D-band^36^. However, the acid treatment can also result in an increase of the amount of CNT structural defects and therefore the relative intensity of the D peak^37^.

Another feature, important for CNT material characterization is the G-band (~1580 cm^−1^). The G-band signature comes from the stretching of carbon-carbon bonds within the graphene plane^7^. Analysis of the G-band may be used to distinguish metallic from semiconducting nanotubes or to examine the electron flow involved with the doping^37^. The G-band is composed of two main components. G+ (~1590 cm^−1^) and G− (~1570 cm^−1^), where the G+ and G− features are related to carbon atom vibrations along and across the nanotube axis respectively. The charge transfer induced by the presence of dopants in the CNT material results in the frequency offset of component G+. The up-shifted G+ frequency indicates the presence of acceptor dopant in the material^38^.

The last feature to be mentioned is the 2D (~2690 cm^−1^), it is the overtone to the D-feature, not related to the disorder^39^.

Changes in absolute intensity of separate features have been reported to be a reliable tool for the analysis of structures composed mostly of SWNTs^34^. In the case of the CNT fibers used in this study, such changes are difficult to observe. Often, as a reliable comparison parameter, the ratio between the intensity of G and D bands is mentioned. However, in CNT fibers composed of a varying content of single-, double- and multi-wall nanotubes, as well as metallic and semiconducting ones, and also considering a multi-step doping process, changes of the ratio parameter are often ambiguous.

A 488 nm laser wavelength was used for the Raman spectra acquisition and the following steps of doping procedure were measured: (1) CNT fibres as made; (2) CNT fibres annealed at 400 °C; (3) CNT fibres annealed and treated with acetone; (4) CNT fibres annealed, treated with acetone and soaked in HCl; (5) fibres annealed, treated with acetone, soaked in HCl and H_2_O_2_ (60%); (6) fibres purified, treated with H_2_O_2_ and doped with 6a- HClO_4_ or 6b- H_2_SO_4_ as well as 6c- purified fibre treated with Br_2_.

**Figure 5:**
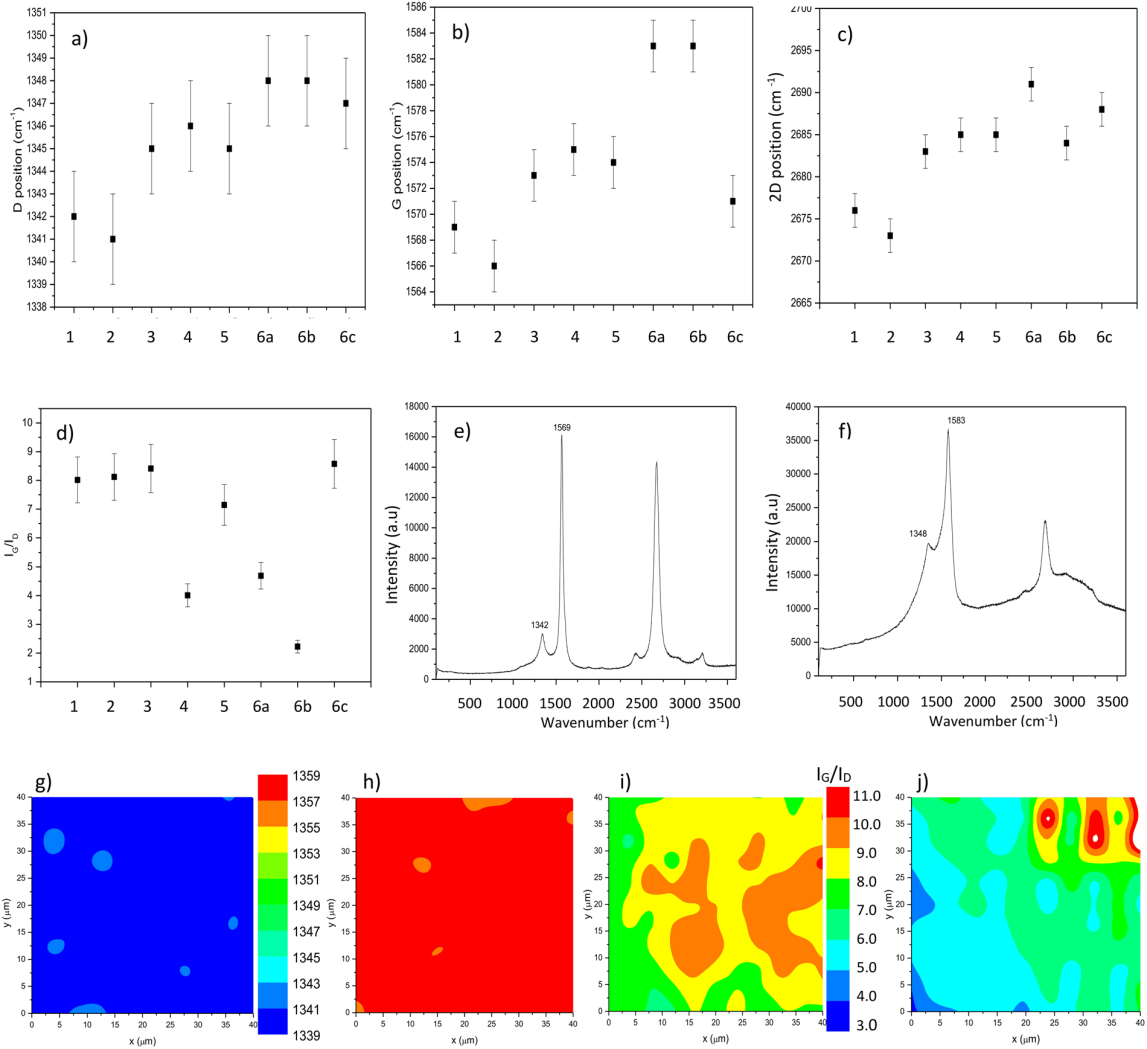
(**a**) Comparison of D- (**b**) G- (**c**) 2D-band position and (**d**) I_G_/I_D_ ratio for all samples (**e**) Raman spectrum for as made and (**f**) H_2_SO_4_ treated CNT wires; Raman maps of D-band position in (**g**) as-made fibre and (**h**) fibre doped with HClO_4_; Raman maps of intensity marker (I_G_/I_D_ ratio) in (**i**) as-made fibre and (**j**) fibre doped with HClO_4_. The error bars in Fig. 5a–c are related to the spectral resolution of the Raman spectrometer. While, in Fig. 5d, with the standard deviation of measurements of the integral intensity of Raman bands.

Considering the results shown in Fig. 5 the following conclusions can be drawn. Annealing (sample 2) involved down shift of all D, G and 2D-band. This behavior may suggest removal of air related dopants, some of which could have had been attracted by amorphous carbon which was partly removed by annealing.

The next steps involved a medium increase of all 3 bands positions, which corresponds with the inferred earlier mild doping of CNT fibres with the chemical compounds used for purification.

Both further acid treatments tested resulted in additional up-shifts of both the D and G band. Whereas, examining the 2D band, H_2_SO_4_ treatment does not cause any shift in the 2D-band position (same shift as for previous, H_2_O_2_ step) but for HClO_4_ there is clear up-shift in the 2D-position. Moreover, the H_2_SO_4_ treatment induces a visible disturbance of the typical Raman spectrum for CNT (Fig. 5f). All of this may again suggest that the sulfuric acid causes a weaker p-doping and a greater destruction of the structure of the CNTs than perchloric acid, which is in agreement with the previously discussed conductivity changes and TGA results. But this may also point at the fact that to recognize the presence of doping, one needs to analyze all of the available peaks.

This method was particularly useful in case of the Br_2_ doping where the G-peak did not show any up-shift while the D and 2D peaks were clearly shifted. Finally, as might have been expected from the previous results, the Br_2_ doped samples did not show any decrease in the I_G_/I_D_ ratio which indicates that sample was not damaged by the Br_2_ treatments.

So as to further check the reliability of the results, Raman mapping on several samples was performed. Figure 5g–j shows the D peaks position and I_G_/I_D_ ratios for as-made and HClO_4_ doped samples. The maps show a very high homogeneity of each of the D, G and 2D peak positions. Figure 5g,h shows the D band position, the same trend was observed for both G and 2D peaks. Yet, the I_G_/I_D_ maps (Fig. 5i,j) exhibited high variability, which could also explain an increased scatter of the data in Fig. 5d.

However, averaging these results over the whole map areas showed a visible shift towards lower ratios for highly oxidized samples, confirming the general trend of a decrease in the I_G_/I_D_ for the oxidized samples.

#### Current carrying capacity

**Figure 6:**
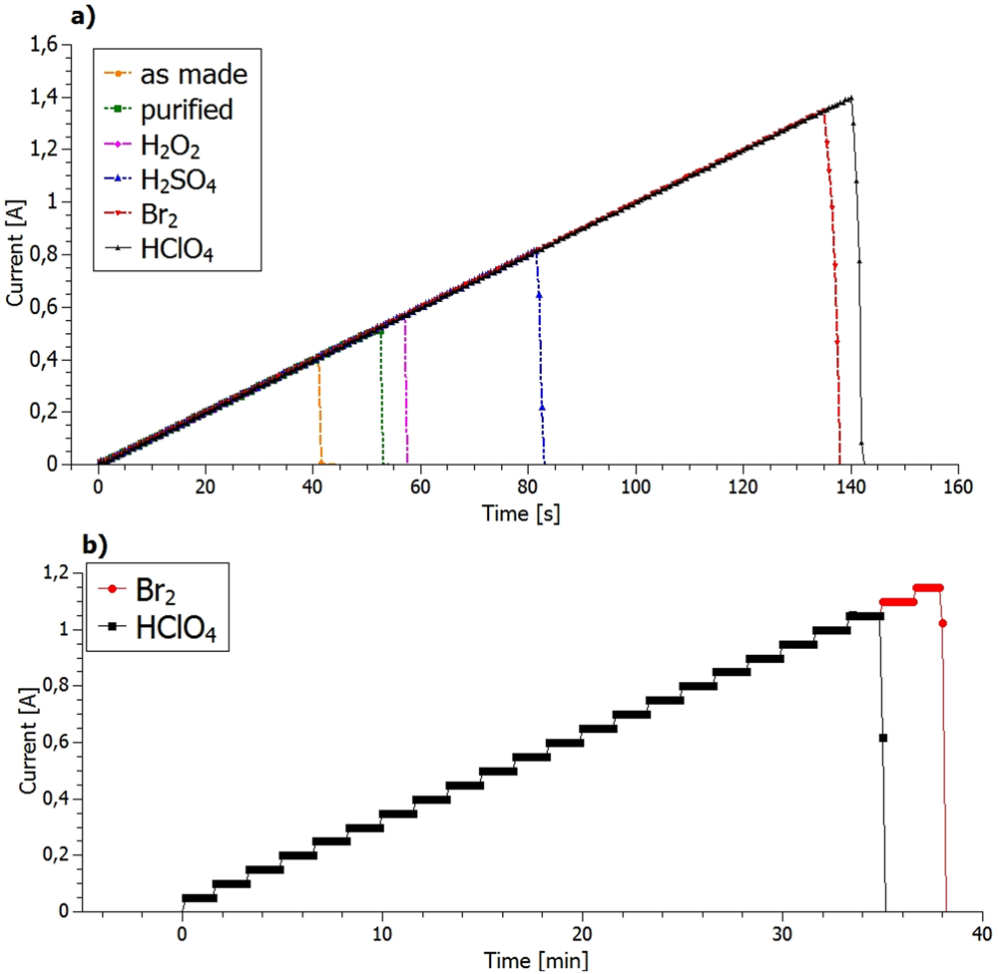
(**a**) CCC measurements comparing samples at different preparation levels and treated with various dopants, tested with smaller current increments of 0.01 A/1 s using smaller steps (current increment step = 0.01 A; step duration = 1 s); (**b**) CCC measurements comparing best dopants tested with larger current increments of 0.5 A/100 s.

The final tests involved the measurement of the current carrying capacity (CCC), which is a very important parameter when considering electrical wires. In this study, a number of tests had been conducted to verify how doping affects the CCC. The current was applied in steps, with a predetermined increment and duration. For comparison of different samples in the same plot, the dependence of the current on time is presented (Fig. 6).

As shown in Fig. 6, after every step of the proposed doping procedure the current that it was possible to transmit through the wire increased. For the three tested dopants, H_2_SO_4_ appeared to be visibly the worst. The best results were obtained for Br_2_ and HClO_4_ (Fig. 6a).

These results are consistent with the electrical conductivity dependencies outlined above.

To verify that the CCC results obtained in the “small increment measurements” were not influenced by their being insufficient time for the full evaporation of the dopants, a second set of measurements were taken, this time with larger increments (current step increased from 0.01 A to 0.5 A, and step duration changed from 1 s to 100 s as compared to previously discussed measurements).

These “longer time measurement” results showed that the HClO_4_ must have only partly evaporated as the final CCC decreased by 25% as compared to small increment measurements. (For bromine doping there was no clear difference in the results compared to the short measurement suggesting its superior performance over the other dopants).

## Conclusions

This research and its associated results allow us to form a clearer picture of both the opportunities and limitations of CNTs fibres operating in harsh oxidizing environments.

The research has facilitated the identification of the most effective purification and pre-treatment methods, which are often used before doping procedures. Interestingly these have been found to also lead to an increase in the effectiveness of further doping. Moreover, measurements have shown that in using these methods the wires can increase in both absolute and specific conductivities by up to 100%.

Further, the tests included doping with a wide range of acids including HCl and oxidizing acids of varying strengths with particular attention paid to the strongest ones as well as with halogens. The acids and halogens were applied in various configurations including multi-compound treatments.

The absolute and specific conductivity measurements were supported by CCC testing, TGA and SEM analyses as well as Raman spectroscopy. These tests showed that the most beneficial doping procedures involved annealing at 400 °C (which partly removes the amorphous carbon), short sonication and soaking in acetone (enabling removal of some hydroxyl functionalities), soaking in HCl (which removed approximately 40 wt.% of catalyst) and using pre-doped fibres.

The above procedures were followed by either Br_2_ doping or H_2_O_2_ soaking (which swelled the fibres to enable better acid penetration) and rarely reported HClO_4_ acid treatment.

These treatments resulted in a very significant doping of the CNT wires, this observed in Raman spectra, and also negligible damage to the structure of the CNT wires at room temperature. In all these produced an increase of approximately 700% in absolute conductivity, an increase of 150% in specific conductivity, and a 341% improvement in current carrying capacity.

However, it has been shown that the action of some of the strongest, hygroscopic and water-reactive acids resulted in the destruction of thinner fibres. Also, treatment by two acids resulted in an increased share of damaged fibres. Comparison of the Raman and TGA features as well as current carrying capacity of sulfur containing acid H_2_SO_4_ with HClO_4_ doped fibres, showed a higher degree of damage to the fibres and a greater sensitivity to temperature related damage caused by H_2_SO_4_ treatment.

**Figure 7:**
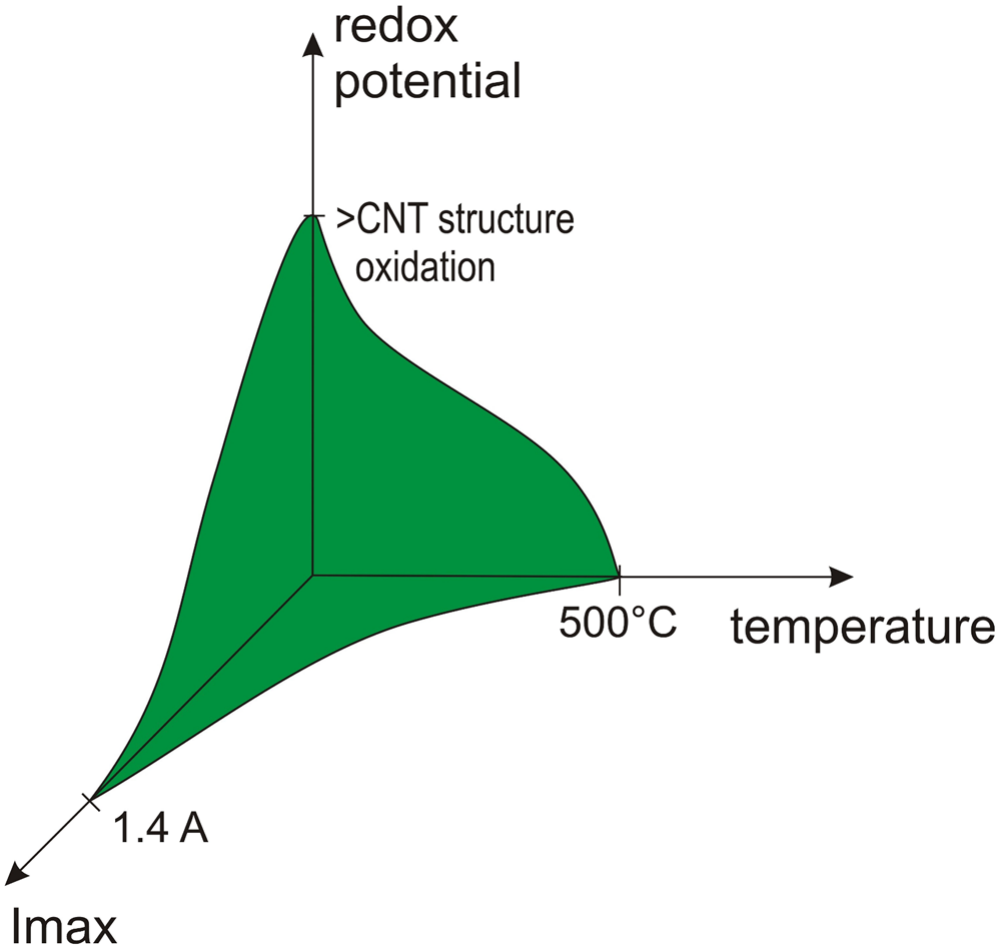
Operational window for CNT wires in strongly oxidizing environments.

This research and the tests performed led to the formulation of the “operational window of the oxidation of CNT wires”. This identifies the factors limiting the performance of the wires in strongly oxidizing environments. These covering temperature, the redox potential of the doping agent and the value of current flowing through the wire (Fig. 7).

Despite the fact that there are identified limits to the performance of CNT wires in harsh oxidation environments, these materials exhibit not only a much higher resistance to such conditions, compared to conventional metals used in electrical wiring, but, against intuition, they may also benefit greatly from their operation in such environments.

This is both an intriguing and a practical fact that should not be neglected when considering the future design of industrial implementations requiring electrical wiring to function successfully, in harsh oxidizing environments.
